# The Distribution and Outcomes of the 21-Gene Recurrence Score in T1-T2N0 Estrogen Receptor-Positive Breast Cancer With Different Histologic Subtypes

**DOI:** 10.3389/fgene.2018.00638

**Published:** 2018-12-17

**Authors:** Jun Wang, Zhen-Yu He, Yong Dong, Jia-Yuan Sun, Wen-Wen Zhang, San-Gang Wu

**Affiliations:** ^1^Department of Radiation Oncology, Xiamen Cancer Hospital, The First Affiliated Hospital of Xiamen University, Xiamen, China; ^2^State Key Laboratory of Oncology in South China, Department of Radiation Oncology, Sun Yat-sen University Cancer Center, Collaborative Innovation Center of Cancer Medicine, Guangzhou, China; ^3^Department of Oncology, Dongguan Third People’s Hospital, Affiliated Dongguan Shilong People’s Hospital of Southern Medical University, Dongguan, China

**Keywords:** breast cancer, Oncotype DX, histologic subtypes, survival, SEER

## Abstract

**Background:** The clinical value of 21-gene recurrence score (RS) in various breast cancer histologic subtypes is not well established.

**Aims:** To assess the distribution and outcomes of the 21-gene RS among various T1-T2N0 estrogen receptor-positive breast cancer histologic subtypes.

**Methods:** Using the Surveillance, Epidemiology and End Results database, we investigated the distribution and outcomes of the 21-gene RS among various breast cancer histologic subtypes between 2004 and 2015. The histologic subtypes with 200 or more cases were further analyzed.

**Results:** We identified 83,665 patients including eight histologic subtypes. The most common subtype was invasive ductal carcinoma not otherwise specified (IDC NOS) (77.9%), followed by lobular carcinoma NOS, mixed infiltrating duct and lobular carcinoma (IDC-L), mucinous adenocarcinoma, tubular adenocarcinoma, micropapillary ductal carcinoma, cribriform carcinoma NOS, and intraductal papillary adenocarcinoma with invasion with 10.8, 7.7, 2.1, 0.6, 0.3, 0.2, and 0.2%, respectively. The 5-years breast cancer specific survival (BCSS) was 98.8, 98.8, 98.9, 99.6, 100, 100, 100, and 100%, respectively (*P* = 0.011). Patients with IDC NOS (8.9%), micropapillary ductal carcinoma (8.8%), and intraductal papillary adenocarcinoma with invasion (8.2%) had significantly higher percentage of high-risk RS compared to other histologic subtypes (1.0–3.8%) (*P* < 0.001). The mean RS was higher in IDC NOS, lobular carcinoma NOS, and IDC-L compared to other subtypes. In multivariate analysis, 21-gene RS was the independent prognostic factor in patients with IDC NOS (*P* < 0.001), lobular carcinoma NOS (*P* < 0.001), and IDC-L (*P* < 0.001), patients with a higher RS was associated with poor BCSS.

**Conclusion:** Our results demonstrate that there is a significant difference in distribution of 21-gene RS in T1-T2N0 estrogen receptor-positive breast cancer with different histologic subtypes. Long-term studies with larger series are needed to confirm the role of the 21-gene RS array in prognosis assessment and chemotherapy decision-making in special histologic subtypes with favorable prognosis.

## Introduction

Breast cancer comprises a heterogeneous cohort of tumors including several histologic subtypes. In patients with hormone-receptor-positive disease, approximately 80% of cases are invasive ductal carcinoma not otherwise specified (IDC NOS) and 15% are invasive lobular carcinoma (ILC). ILC has favorable prognostic factors including higher hormone receptor-positive, lower tumor grade as well as lower proliferation rates than IDC (Pestalozzi et al., 2008; Rakha et al., 2008a,b; Rakha and Ellis, 2010; Loibl et al., 2014). However, long-term results indicated that the ILC had poor outcomes compared to IDC NOS subtype (Rakha and Ellis, 2010; Loibl et al., 2014). The other special histologic subtypes are rare and show variation in their prognosis and response to treatment. Mucinous, papillary and tubular carcinomas are known to have an extremely favorable prognosis with significantly lower recurrence rates compared with IDC NOS and ILC subtypes (Page, 2003; Li et al., 2005; Pestalozzi et al., 2008; Yerushalmi et al., 2009; Weigelt et al., 2010; Lakhani et al., 2012; Nagao et al., 2012). Since there are currently no prospective studies of specific treatment decisions for the special histologic subtypes, optimal management strategies require further investigation.

The 21-gene Oncotype DX Breast Recurrence Score (RS) assay (Genomic Health, Redwood City, CA, United States) is a reverse-transcriptase polymerase chain reaction test which measures the expression of 16 breast cancer genes and 5 reference genes. A RS is then used to assign patients into one of three prognostic groups including low-risk (RS < 18), intermediate-risk (RS 18–30) or high-risk (RS > 30) (Paik, 2007; Wong et al., 2012). This widely validated gene-expression profiles has been used to evaluate the probability of distant metastasis at 10 years and adjuvant chemotherapy decision-making in early-stage estrogen receptor (ER)-positive, node-negative invasive breast cancers (Paik et al., 2004, 2006; Mamounas et al., 2010; Petkov et al., 2016; Krop et al., 2017; Sparano et al., 2018). However, those prospective and population-based studies did not distinguish results between the various histologic subtypes. Therefore, the clinical value of this assay in predicting outcomes of various subtypes is not well established. It is not clear whether the results from one subtype can be generalized to other subtypes. In this study, we investigated the distribution and outcomes of the 21-gene RS assay among various T1-T2N0 ER-positive breast cancer histologic subtypes using a population-based registry database.

## Materials and Methods

### SEER Database and Patients

We analyzed breast cancer patients between 2004 and 2015 using the Surveillance, Epidemiology and End Results (SEER) program. The SEER dataset is maintained by the National Cancer Institute and consists of de-identified cancer incidence and survival data from 18 cancer registries across United States (Surveillance Epidemiology End Results [SEER], 2018). We included subtypes of node-negative and ER-positive disease where the tumor size was ≤ 5 cm (T1-2 stage) according to the International Classification of Diseases for Oncology, 3rd edition (Fritz et al., 2000), and the results of 21-gene RS assay were available. We only included subtypes with >200 cases. Exclusion criteria were: unknown surgical procedure, no pathological diagnosis and unknown status of tumor grade. Using data from SEER was exempt from the approval process of Institutional Review Boards due to the de-identification of patients.

### Variables

We extracted the following variables from the SEER dataset: age, race/ethnicity, tumor grade, histologic subtypes, tumor stage, progesterone receptor (PR) status, surgical procedure, radiotherapy, chemotherapy and 21-gene RS. The groups of 21-gene RS were defined as low-risk (RS < 18), intermediate-risk (RS 18–30) or high-risk (RS > 30) (Paik, 2007; Wong et al., 2012). The primary survival outcome of this study was breast cancer-specific survival (BCSS).

### Statistical Analysis

The relationships between the histologic subtypes, patient demographics, clinicopathological characteristics and treatment were compared with chi-squared or Fisher’s exact probability tests. ANOVA was used to compare continuous variables. Kaplan-Meier curves were plotted and compared using the log-rank test in patients diagnosed between 2004 and 2012. The independent prognostic factors related to BCSS in patients diagnosed between 2004 and 2012 were analyzed using multivariate Cox proportional hazards models. All analyses were conducted with SPSS Statistical Software, version 22 (IBM Corporation, Armonk, NY, United States), and *P*-values of < 0.05 were considered statistically significant.

## Results

**FIGURE 1:**
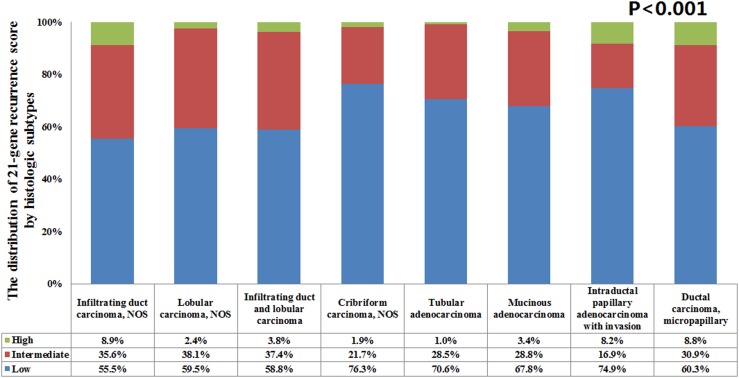
The distribution of 21-gene recurrence score among histologic subtypes of breast cancer.

We identified 83,665 patients in this study including 8 histologic subtypes. The most common histologic subtype was IDC NOS (*n* = 65,179; 77.9%) followed by lobular carcinoma (NOS; *n* = 9037; 10.8%), mixed infiltrating duct and lobular carcinoma (IDC-L; *n* = 6483; 7.7%), mucinous adenocarcinoma (*n* = 1760; 2.1%), tubular adenocarcinoma (*n* = 520; 0.6%), micropapillary ductal carcinoma (*n* = 272; 0.3%), cribriform carcinoma NOS (*n* = 207; 0.2%), and intraductal papillary adenocarcinoma with invasion (*n* = 207; 0.2%). The patient characteristics according to histologic subtypes are shown in Table 1. Low-, intermediate- and high-risk RS were seen in 47,405 (56.7%), 29,868 (35.7%), and 6392 (7.6%) patients, respectively. Significantly higher percentages of high-risk RS were seen in patients with IDC NOS (8.9%), micropapillary ductal carcinoma (8.8%) and intraductal papillary adenocarcinoma with invasion (8.2%) compared to other histologic subtypes (1.0–3.8%) (*P* < 0.001) (Figure 1). These were significantly different in the mean RS of various histologic subtypes (*P* < 0.001). The mean RS in IDC NOS, lobular carcinoma NOS, IDC-L, cribriform carcinoma NOS, tubular adenocarcinoma, mucinous adenocarcinoma, intraductal papillary adenocarcinoma with invasion and micropapillary ductal carcinoma were 18 (range, 0–100), 16 (range, 0–55), 17 (range, 0–65), 12 (range, 0–37), 15 (range, 1–45), 15 (range, 0–59), 12 (range, 0–53), and 16 (range, 0–52), respectively.

**Table 1:** Patient characteristics by histologic subtypes.

Variables	*n*	IDC NOS (%)	Lobular carcinoma NOS (%)	IDC-L (%)	Cribriform carcinoma NOS (%)	Tubular adenocarcinoma (%)	Mucinous adenocarcinoma (%)	Intraductal papillary adenocarcinoma with invasion (%)	Micropapillary ductal carcinoma (%)	*P*
Age (mean ± SD) (years)	59 ± 10.5	57.9 ± 10.5	60.1 ± 9.9	58.3 ± 10.0	56.6 ± 11.9	55.5 ± 9.7	58.5 ± 12.1	62.1 ± 10.8	60.0 ± 10.7	<0.001
**Race/ethnicity**									
Non-Hispanic white	62,665	48,623 (74.6)	6984 (77.3)	5026 (77.6)	147 (71.0)	409 (78.7)	1161 (66.0)	136 (65.7)	179 (65.8)	<0.001
Non-Hispanic black	6326	4902 (7.5)	731 (8.1)	369 (5.7)	19 (9.2)	42 (8.1)	203 (11.5)	31 (15.0)	29 (10.7)	
Hispanic (all races)	7096	5502 (8.4)	727 (8.0)	607 (9.4)	16 (7.7)	44 (8.5)	159 (9.0)	14 (6.8)	27 (9.9)	
Other	7578	6152 (9.4)	595 (6.6)	481 (7.4)	25 (12.1)	25 (4.8)	237 (13.5)	26 (12.6)	37 (13.6)	
**Grade**									
Well differentiated	23,803	17,970 (27.6)	2641 (29.2)	1496 (23.1)	119 (57.5)	478 (91.9)	1008 (57.3)	69 (33.3)	22 (8.1)	<0.001
Moderately differentiated	45,786	34,796 (53.4)	5770 (63.8)	4141 (63.9)	77 (31.2)	37 (7.1)	695 (39.5)	104 (50.2)	166 (61.0)	
Poorly/undifferentiated	14,076	12,413 (19.0)	626 (6.9)	846 (13.0)	11 (5.3)	5 (1.0)	57 (3.2)	34 (16.4)	87 (32.0)	
**Tumor stage**									
T1	65,284	52,394 (80.4)	5857 (64.8)	4869 (75.1)	162 (78.3)	497 (95.6)	1154 (65.6)	151 (72.9)	200 (73.5)	<0.001
T2	18381	12,785 (19.6)	3180 (35.2)	1614 (24.9)	45 (21.7)	23 (4.4)	606 (34.4)	56 (27.1)	72 (26.5)	
**PR status (*n* = 83,510)**									
Negative	8102	6123 (9.4)	1161 (12.9)	598 (9.3)	10 (4.9)	44 (8.5)	120 (6.9)	24 (11.6)	22 (8.1)	<0.001
Positive	75,408	58,951 (90.6)	7860 (87.1)	5865 (90.7)	196 (95.1)	472 (91.5)	1631 (93.1)	183 (88.4)	250 (91.9)	
**Surgery procedure**									
Breast conserving surgery	56,701	45,309 (69.5)	5295 (58.6)	4063 (62.7)	135 (65.2)	385 (74.0)	1217 (69.1)	125 (60.4)	172 (63.2)	<0.001
Mastectomy	26,964	19,870 (30.5)	3742 (41.4)	2420 (37.3)	72 (34.8)	135 (26.0)	543 (30.9)	82 (39.6)	100 (36.8)	
**Radiotherapy**									
No/unknown	34,715	26,225 (40.2)	4308 (47.7)	2955 (45.6)	91 (44.0)	193 (37.1)	726 (41.3)	96 (46.4)	121 (44.5)	<0.001
Yes	48,950	38,954 (59.8)	4729 (52.3)	3528 (54.4)	116 (56.0)	327 (62.9)	1034 (58.7)	111 (53.6)	151 (55.5)	
**Chemotherapy**									
No/unknown	67,288	51,603 (79.2)	7696 (85.2)	5385 (83.1)	184 (88.9)	476 (91.5)	1533 (87.1)	182 (87.9)	229 (84.2)	<0.001
Yes	16,377	13,576 (20.8)	1341 (14.8)	1098 (16.9)	23 (11.1)	44 (8.5)	227 (12.9)	25 (12.1)	43 (15.8)	
**21-Gene recurrence score**									
Low-risk	47,405	36,181 (55.5)	5374 (59.5)	3813 (58.8)	158 (76.3)	367 (70.6)	1193 (67.8)	155 (74.9)	164 (60.3)	<0.001
Intermediate-risk	29,868	23,184 (35.6)	3443 (38.1)	2422 (37.4)	45 (21.7)	148 (28.5)	507 (28.8)	35 (16.9)	84 (30.9)	
High-risk	6392	5814 (8.9)	220 (2.4)	248 (3.8)	4 (1.9)	5 (0.9)	60 (3.4)	17 (8.2)	24 (8.8)	

*IDC-L, mixed infiltrating duct and lobular carcinoma; IDC NOS, invasive ductal carcinoma not otherwise specified; PR, progesterone receptor; SD, standard deviation; T, tumor.*

**FIGURE 2:**
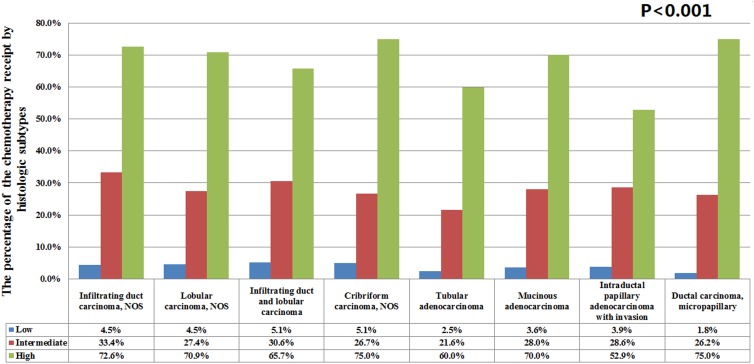
The percentage of chemotherapy administration among histologic subtypes of breast cancer.

A total of 16,337 (19.5%) patients received chemotherapy. The percentage of chemotherapy administration was related to the results of 21-gene RS assay. A total of 4.5, 32.3, and 72.2% of patients with low-, intermediate-, and high-risk RS were treated with chemotherapy, respectively (*P* < 0.001). The percentage of chemotherapy administration was not significantly different in low- (*P* = 0.093) and high-risk RS (*P* = 0.073) across the various histologic subtypes. However, patients with tubular carcinoma and intermediate RS had a significantly lower percentage of chemotherapy receipt than other histologic subtypes (21.6% vs. 26.2–33.4%; *P* < 0.001) (Figure 2).

A total of 50,660 patients were diagnosed between 2004 and 2012 with a median follow-up period of 65 months (range, 0–143 months). Of these patients, 784 patients had died with breast cancer related disease. Patients with a higher RS were related to poor BCSS, the 5-years BCSS in patients with low-, intermediate-, and high-risk RS were 99.5, 98.6, and 95.2%, respectively (*P* < 0.001). In patients with IDC NOS, lobular carcinoma NOS, IDC-L, cribriform carcinoma NOS, tubular adenocarcinoma, mucinous adenocarcinoma, intraductal papillary adenocarcinoma with invasion and micropapillary ductal carcinoma, the 5-years BCSS was 98.8, 98.8, 98.9, 100, 100, 99.6, 100, and 100%, respectively (*P* = 0.011); and a total of 644, 73, 58, 2, 1, 6, 0, and 0 patients, respectively, died from breast cancer-related disease.

**FIGURE 3:**
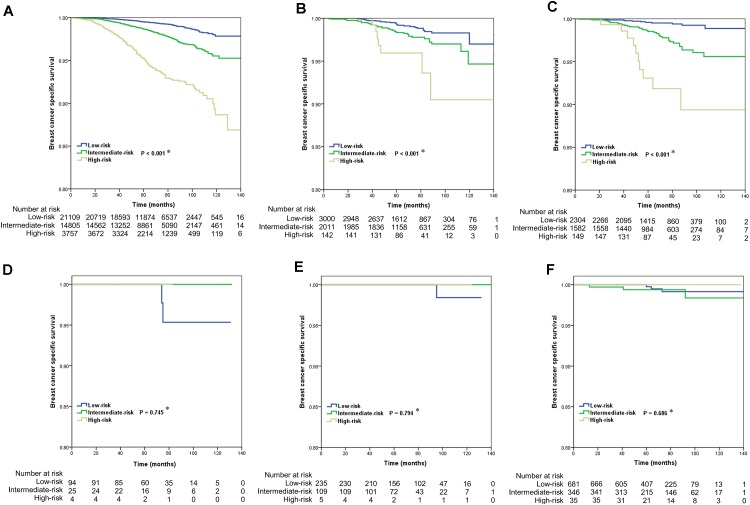
Kaplan-Meier curves of the effect of 21-gene recurrence score on breast cancer specific survival by histologic subtypes of breast cancer (**A**, IDC NOS; **B**, lobular carcinoma NOS; **C**, IDC-L; **D**, cribriform carcinoma NOS; **E**, tubular adenocarcinoma; **F**, mucinous adenocarcinoma) (^∗^ low-risk vs. intermediate-risk vs. high-risk).

The results of the Kaplan-Meier curves are shown in Figure 3. In patients with IDC NOS (*P* < 0.001) (Figure 3A), lobular carcinoma NOS (*P* < 0.001) (Figure 3B) and IDC-L (*P* < 0.001) (Figure 3C), a higher RS was significantly associated with poor BCSS. However, the 21-gene RS was not related to BCSS in patients with cribriform carcinoma NOS (*P* = 0.745) (Figure 3D), tubular adenocarcinoma (*P* = 0.794) (Figure 3E), and mucinous adenocarcinoma (*P* = 0.686) (Figure 3F). The survival curves of intraductal papillary adenocarcinoma with invasion and micropapillary ductal carcinoma subtypes could not be applied because no patients died during follow-up.

When adjusted for age, race/ethnicity, grade, tumor stage, PR status, surgical procedure, radiotherapy, and chemotherapy, a higher RS was independently related to poor BCSS in patients with IDC NOS [hazard ratio (HR) 2.159, 95% confidence interval (CI) 1.925–2.420, *P* < 0.001], lobular carcinoma NOS (HR 2.139, 95% CI 1.480–3.093, *P* < 0.001) and IDC-L (HR 3.164, 95% CI 2.097–4.776, *P* < 0.001) (Table 2). Due to the low number of breast cancer-specific deaths, we did not perform multivariate prognostic analysis of the remaining five histologic subtypes.

**Table 2:** Multivariate analysis for prognostic factors of breast cancer-specific survival by histologic subtypes.

Variables	HR	95% CI	*P*
**IDC NOS**			
Age	1.020	1.013–1.028	<0.001
Race/ethnicity	0.976	0.897–1.061	0.563
Grade	1.669	1.464–1.902	<0.001
Tumor stage	1.910	1.619–2.253	<0.001
PR status	0.982	0.800–1.205	0.861
Surgery procedure	0.922	0.741–1.146	0.463
Radiotherapy	0.691	0.591–0.807	<0.001
Chemotherapy	0.969	0.808–0.164	0.739
21-gene recurrence score	2.159	1.925–2.420	<0.001
**Lobular carcinoma NOS**			
Age	1.024	1.000–1.050	0.051
Race/ethnicity	0.967	0.735–1.274	0.813
Grade	1.380	0.918–2.073	0.121
Tumor stage	3.490	2.139–5.695	<0.001
PR status	0.805	0.455–1.425	0.456
Surgery procedure	1.218	0.641–2.315	0.547
Radiotherapy	0.478	0.291–0.785	0.004
Chemotherapy	1.084	0.615–1.910	0.781
21-gene recurrence score	2.139	1.480–3.093	<0.001
**IDC-L**			
Age	1.037	1.009–1.065	0.008
Race/ethnicity	0.885	0.631–1.241	0.480
Grade	1.488	0.955–2.321	0.079
Tumor stage	2.281	1.345–3.870	0.002
PR status	0.854	0.444–1.642	0.635
Surgery procedure	0.964	0.484–1.918	0.916
Radiotherapy	0.717	0.361–1.426	0.343
Chemotherapy	0.911	0.489–1.696	0.768
21-gene recurrence score	3.164	2.097–4.776	<0.001

*CI, confidence interval; HR, hazard ratios; IDC-L, mixed infiltrating duct and lobular carcinoma; IDC NOS, invasive ductal carcinoma not otherwise specified; PR, progesterone receptor.*

## Discussion

Most previous studies involving the 21-gene RS were from invasive breast carcinoma NOS or other subtypes dominated by invasive ductal carcinoma (Mamounas et al., 2010; Albanell et al., 2016; Barcenas et al., 2017; Stemmer et al., 2017; Turashvili et al., 2017b; Sparano et al., 2018). Our results also confirmed that approximately 95% of patients were of IDC and/or ILC subtypes, while the remaining 5% were special histologic subtypes, including cribriform, mucinous, tubular, and papillary carcinomas. Due to the rarity of these subtypes, little is known about the variability of the 21-gene RS results (Hou et al., 2017; Turashvili et al., 2017a). Our results investigated the distribution and survival outcome of different histologic subtypes to determine the clinical practice of 21-gene RS in breast cancer, particularly for special histologic subtypes.

Two recent studies with large cohorts have assessed the distribution of 21-gene RS in invasive breast carcinoma. One study with 610,350 patients (80.9% node-negative) from the Genomic Health Clinical Laboratory found that the mean RS in IDC NOS, lobular carcinoma NOS, IDC-L, mucinous, papillary, tubular, and cribriform carcinoma were similar to our study (Tadros et al., 2018). The distribution of 21-gene RS by various histologic subtypes was also similar. Another population-based study from SEER with 45,618 patients also showed a similar distribution of RS in IDC, ILC, IDC-L, tubular, and mucinous carcinoma (Kizy et al., 2018). However, this study differs from ours in two aspects: first, they did not include papillary and cribriform carcinoma; and secondly they included patients with tumor size >5 cm and whose disease was node-positive. This is in contrast to the recommendation for the 21-gene RS test in breast cancer, which is for patients with small tumors, node-negative, and ER-positive disease (Partin and Mamounas, 2011; Krop et al., 2017; Sparano et al., 2018). In this study, we only included patients with T1-2N0 and ER-positive disease, which represents the current clinical practice. Our study added the current knowledge of the distribution of 21-gene RS in patients with special histologic subtypes.

The main finding of the current study was the significant diversity in the RS results of patients with different histologic subtypes. Our study used a large cohort of patients from a population-based registered database with standardized results of the 21-gene RS test. We found that mean RS results from special breast cancer histologic subtypes were lower than in patients with IDC, ILC and IDC-L subtypes. However, high RS were found in approximately 8% of patients with papillary or micropapillary subtype, which was comparable with IDC NOS but significantly higher than other subtypes, and similar to results from the Genomic Health Clinical Laboratory (Tadros et al., 2018). This suggests that different breast cancer histologic subtypes may have biological diversity.

The 21-gene RS is related to risk of recurrence and administration of chemotherapy. However, there are few outcome data specific to histologic subtypes. Our population-based study showed that a higher RS was related to poor BCSS in IDC NOS, lobular carcinoma NOS and IDC-L. In addition, a higher RS was related to a higher percentage of receiving chemotherapy. We also found the 21-gene RS may impact the decision-making of chemotherapy in patients with special histologic subtypes. However, the short follow-up time and the very low number of deaths for these special histologic subtypes limited our study to reach a definitive conclusion regarding to the outcomes by 21-gene RS groups. Our results raise the question of whether the results of the 21-gene RS are necessary for favorable breast cancer histologic subtypes.

ILC presents with a different pattern of invasion and recurrence compared to IDC NOS, and with a poor 10-years prognosis (Rakha and Ellis, 2010; Loibl et al., 2014). Moreover, ILC has a higher probability of hormone receptor-positive status, a higher probability of lower tumor grade and lower proliferation rates than IDC (Pestalozzi et al., 2008; Rakha et al., 2008a,b; Rakha and Ellis, 2010; Loibl et al., 2014). Therefore, the utility of the 21-gene RS test in ILC requires further investigation. A study by Conlon et al. (2015) found that RS played an important role in chemotherapy decision-making in ILC. Kizy et al. suggested that a high-risk RS was independently related to poor outcome compared to a low-risk RS. However, the adjuvant chemotherapy was not associated with a survival benefit in intermediate- or high-risk patients (Kizy et al., 2017). Our study also confirmed that RS has prognostic value in ILC, but also affects chemotherapy decision-making for patients. A similar trend was seen in patients with IDC-L.

Until now, very few studies have investigated the clinical value of 21-gene RS in special histologic subtypes. One study on 57 patients with special subtypes (including tubular, mucinous, solid papillary and encapsulated papillary carcinoma) found that most were low-risk RS disease, and none had distant metastasis after a median follow-up of 40 months (Turashvili et al., 2017a). In our study, approximately 30 and 70% of patients with intermediate- and high-risk RS received chemotherapy, respectively. In the current clinical practice, histologic subtype is not an important indicator in the decision-making of chemotherapy for patients with different 21-gene RS groups. Several studies have shown that patients receiving chemotherapy have a direct increase in the risk of cognitive complaints and cardiovascular toxicity (Ganz et al., 2013; Guo and Wong, 2014). Therefore, when there is significant heterogeneity within tumors of invasive breast cancer, the 21-gene RS test may be deferred. A multidisciplinary team should discuss whether to perform this test in special breast cancer histologic subtypes.

We acknowledge several limitations in our study. First, there are inherent biases in any retrospective analysis. Secondly, as the median follow-up period in our cohort was only 65 months, longer-term results are required to draw definitive conclusions on the use of 21-gene testing in prognosis and chemotherapy decision-making. In addition, although we included a large sample of patients from a cancer registry database, only a limited number of patients were classified as special histologic subtypes, which may have potential impact on statistical power when comparing the outcome of 21-gene RS groups in these cohorts. Finally, it has been indicated that there are many inaccuracies in the SEER data registry, with a high rate of under-recording chemotherapy administration (Noone et al., 2016). Nevertheless, despite the short follow-up, the BCSS was close to 100% as expected.

## Conclusion

In conclusion, our results demonstrate that there is a major difference in distribution of 21-gene RS among breast cancer histologic subtypes. Long-term studies with a larger series are needed to confirm the role of the 21-gene RS array in prognosis assessment and chemotherapy decision-making in special histologic subtypes with favorable prognosis.

## Author Contributions

S-GW aided in data collection, data analysis, tables and figures creation, and manuscript revision. JW, Z-YH, YD, and J-YS aided in drafting the manuscript and manuscript revision. W-WZ and S-GW are the corresponding authors who initially developed the concept and drafted and revised the manuscript. All authors read and approved the final manuscript.

## Conflict of Interest Statement

The authors declare that the research was conducted in the absence of any commercial or financial relationships that could be construed as a potential conflict of interest.
